# Re-establishing bile acid composition after treatment of recurrent *Clostridioides difficile* infection with fecal microbiota transplantation compared with oral vancomycin or a 12-strain bacterial mixture

**DOI:** 10.1080/19490976.2026.2658915

**Published:** 2026-04-17

**Authors:** Anne Abildtrup Rode, Henri Duboc, Antonin Lamazière, Dominique Rainteau, Lydie Humbert, Emilie Gauliard, Mahtab Chehri, Andreas Munk Petersen, Morten Helms, Kristian Schønning, Henrik Vedel Nielsen, Peter Bytzer, Jørgen Engberg

**Affiliations:** a Department of Medicine, Zealand University Hospital, Koege, Denmark; b Université Paris Cité, CRI Inserm UMRS 1149, équipe PIMS, Paris, France; c AP-HP, Hopital Louis Mourier, DMU ESPRIT, Department of Gastroenterology, Colombes, France; d Sorbonne Université, Inserm, Centre de Recherche Saint-Antoine, CRSA, AP-HP, Hôpital Saint Antoine, Clinical Metabolomics Department, Paris, France; e Department of Infectious Diseases, Hvidovre University Hospital, Hvidovre, Denmark; f Department of Clinical Medicine, University of Copenhagen, Copenhagen, Denmark; g Department of Gastroenterology, Hvidovre University Hospital, Hvidovre, Denmark; h Department of Clinical Microbiology, Hvidovre University Hospital, Hvidovre, Denmark; i Department of Clinical Microbiology, Rigshospitalet, Copenhagen University Hospital, Copenhagen, Denmark; j Department of Bacteria, Parasites & Fungi, Statens Serum Institut, Copenhagen, Denmark; k Department of Clinical Microbiology, Zealand University Hospital, Slagelse, Denmark

**Keywords:** Bile acid homeostasis, microbiota manipulation, infectious colitis, bacterial consortium

## Abstract

Patients with *Clostridioides difficile* infection have high colonic levels of primary bile acids, which are potent germinators of *Clostridioides difficile.* Several studies have suggested that re-establishing a normal bile acid composition is a key factor in fecal microbiota transplantation (FMT) for recurrent *C. difficile* infection, yet former studies supporting this lacked controls. In a subgroup from a randomized controlled trial, we compared the bile acid composition in patients with recurrent *C. difficile* infection treated with either FMT, a bacterial mixture, or vancomycin. The fecal bile acid content was analyzed several times before and after treatments. Furthermore, we used 16S rDNA gene sequencing to analyze the presence of some bacterial species involved in bile acid metabolism. Stool donors served as healthy controls. We observed a higher proportion of primary bile acids in patients with recurrent *C. difficile* infection than in donors, yet a donor-like dominance of secondary bile acids was observed after successful treatment in all groups. The shift seemed to occur earliest in the FMT group, followed by the vancomycin group, and the latest in the bacterial mixture group. In approximately half of the participants, the rise in secondary bile acids was timely associated with the detection of bile acid-transforming bacteria that were absent before treatment. Our findings indicate that FMT re-establishes the bile acid composition faster than vancomycin, reducing the time of susceptibility to recurrences of *C. difficile* infection. Hence, bacterial mixtures developed as an alternative to donor stool for treating recurrent *C. difficile* infection might benefit from including bile acid-metabolizing bacteria.

## Introduction

Fecal microbiota transplantation (FMT) following a pre-treatment with oral vancomycin is the most effective treatment for recurrent *Clostridioides difficile* infections, with cure rates as high as 76%–92%.^1^^-^^4^ However, the mechanism(s) of action behind this effect are largely unknown. One of the main theories is that FMT re-establishes the normal colonic bile acid composition, leading to a re-established resistance towards *C. difficile* infection.^5^

In the gut, conjugated primary bile acids are excreted into the small intestine. Here, several different bacteria that produce bile salt hydrolases are involved in the deconjugation process, whereafter the bile acids are reabsorbed. However, a small proportion reaches the colon, where the deconjugated primary bile acids are transformed into secondary bile acids through 7α-dehydroxylation – a process performed by only a few different bacteria within the *Clostridium* genus, e.g., *C. scindens*, *C. hylemonae, C. hiranonis, C. leptum*, and *C. sordellii.*^6^^-^^9^ Thus, in healthy individuals, mostly secondary bile acids such as lithocholic acid (LCA) and deoxycholic acid (DCA) are present in the colon (approximately 90% of the total bile acids), while the primary bile acids, such as chenodeoxycholic acid (CDCA) and cholic acid (CA), are present at very low concentrations.^6^^-^^8^ *C. difficile* germinates from spores to active vegetative toxin-producing bacteria in the presence of certain primary bile acids in the gut. On the other hand, most secondary bile acids inhibit the germination and growth of *C. difficile.*^6-8^^,^^10-12^ Thus, the colonic bile acid composition in healthy individuals yields an environment unfavorable for *C. difficile* germination and growth.^12^ On the contrary, high levels of primary bile acids and decreased levels of secondary bile acids have been observed in stool from persons exposed to antibiotics and patients with *C. difficile* infection,^10^^,^^12-14^ yielding a colonic environment favoring *C. difficile*.

Studies investigating bile acids in the stool from patients with recurrent *C. difficile* infection receiving FMT have consistently reported a shift from an impaired bile acid composition to a composition resembling healthy stool donors.^15^^-^^22^ This has led to the hypothesis that FMT re-introduces bacteria with 7α-dehydroxylation activity, leading to a recovery of the colonic bile acid composition with a net effect of inhibiting *C. difficile* and preventing new recurrences. Despite the concordance of these findings, the studies have major limitations. The study populations are small, the timing of sampling is heterogenic, and bile acids are often measured very late after FMT (up to 3–6 months after FMT, i.e., much later than the natural reestablishment of bile acids would be expected after exposure to antibiotics).^15^^-^^23^ Most importantly, all studies lack control groups. Thus, we are unaware of whether the recovery of the bile acid composition is a result of FMT or merely a result of natural re-establishment after any successful treatment for *C. difficile* infection.

Here, we present a sub-study nested in a randomized controlled trial investigating the effect of FMT for recurrent *C. difficile* infection compared with either a well-characterized 12-strain bacterial mixture or standard treatment with oral vancomycin as controls. The results from the randomized controlled trial have been reported elsewhere.^4^ In this sub-study, we examined the development of the bile acid composition in stool samples from several time points after the three treatments. Furthermore, using 16S rDNA gene sequencing, we investigated if the presence of bacterial species capable of performing 7α-dehydroxylation was associated with this development.

We hypothesized that treatment with FMT and the bacterial mixture would re-establish a normal bile acid composition faster and to a larger extent than after vancomycin monotherapy (with a normal composition defined as a composition dominated by secondary bile acids as seen in healthy individuals).^6^^-^^8^

## Materials and methods

### Study design and population

**Figure 1.**
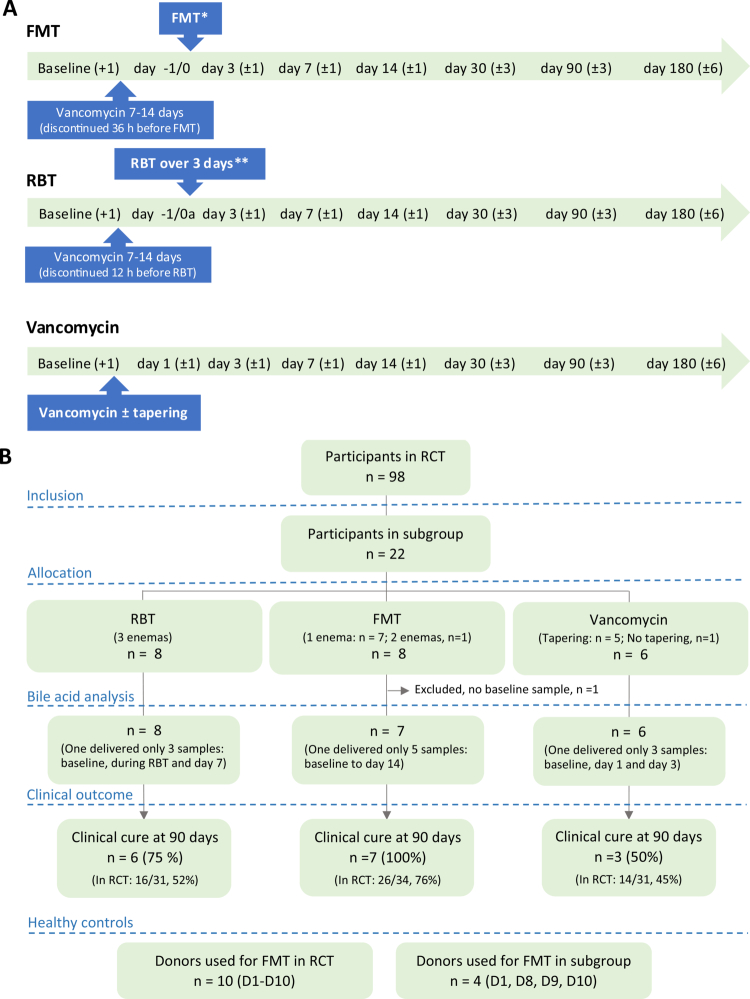
(A) Trial interventions and sampling times for the three treatment groups. Trial interventions are shown for each group. Pretreatment before FMT and RBT includes 7–14 d of oral vancomycin 125 mg four times a day. FMT was given 1–3 times according to predefined clinical criteria.^4^ The Vancomycin group received oral vancomycin 125 mg four times a day for a minimum of 14 d with additional tapering (125 mg twice a day for 1 week, 125 mg once daily for 1 week, 125 mg every other day for 1 week, and 125 mg every third day for 2 weeks) for patients with multiple recurrences. Baseline (+1) = on the day of inclusion or the day after (possible prior to the intake of vancomycin for 0–7 d). Day -1/0 = the day before or on the day of beginning FMT/RBT, i.e., after 7–14 d pre-treatment with oral vancomycin. Day 1–180 =  days after ended treatment, i.e., starting the day after (last) FMT, after the third RBT, or after the last intake of oral vancomycin with or without tapering. *In the case of repeat-FMT sampling started over on day 3 after repeat-FMT. **Day 0a = the first day of the three RBT infusion days, day 3 = 3 d after the third RBT-infusion. (B) Flowchart of the study population/material and clinical effects of the three treatments. FMT: fecal microbiota transplantation; RBT: Rectal bacteriotherapy. RCT: randomized controlled trial, see details in reference.^4^

We included a subgroup of the participants from a two-center open-label randomized controlled trial. This trial compared the efficacy of three treatments for patients with recurrent *C. difficile* infection, i.e., FMT *vs*. standard treatment with oral vancomycin *vs*. a defined mixture of 12 enteric bacteria termed rectal bacteriotherapy.^4^ The trial interventions are shown in Figure 1A. The bacterial strains included in the mixture for rectal bacteriotherapy are shown in Table 1 together with the main characteristics of the stool donors used for FMT. The development of the bacterial mixture^24^^,^^25^ and the process of screening and testing donors^26^ have been described elsewhere. The primary endpoint of the main trial was clinical cure, defined as the absence of *C. difficile* infection within 90 d after ended treatment.

**Table 1.** The composition of bacterial strains in the rectal bacteriotherapy mixture.^24^

‐ **Escherichia coli*, MT-1108-1* ‐ **Escherichia coli*, MT- 1109* ‐ **Enterococcus cassiliflavus* ^*1*^* ‐ **Enterococcus gallinarum* ^*2*^* ‐ *Bacteroides thetaiotaomicron* ‐ *Bacteroides ovatus* ‐ *Bacteroides vulgatus* ‐ *Clostridium bifermentans* ‐ *Clostridium innocuum* ‐ **Coprobacillus cateniformis* ^*3*^* ‐ *Lactobacillus rhamnosus* ‐ **Lactobacillus gasserii* ^*4*^*		
*Baseline characteristics of stool donors used for FMT (n = 10)*		
Women, *n* (%)	6 (60%)	
Age in years, median (IQR; range)	41.5 (26.8–48.8; 21–63)	
BMI, median (IQR; range)	24.2 (22.1–24.9; 19.9–25.0)	

^1^^-^^4^ Formerly identified as (1) *Enterococcus faecalis*, (2) *Enterococcus faecium*, (3) *Clostridium rhamnosum,* and (4) *Lactobacillus acidophilus.* FMT: fecal microbiota transplantation; BMI: Body mass index; IQR: Interquartile range.

We selected the subgroup for bile acid analyses based on pragmatic criteria to ensure sufficient compliance with the comprehensive sampling plan. The participants had to be able to collect and store stool samples at home according to instructions – possibly with the help of relatives or dedicated caregivers. Hence, given the frailty of many of the participants, we did not see it as possible to randomly select participants for the subgroup. The participants were selected before randomization.

### Sampling

We collected eight stool samples from each participant, with the sample times distributed before and after treatment over the 6-month study period, Figure 1A. Of note, the three treatment courses differed somewhat, including the timing of vancomycin discontinuation, leading to some inherent differences in the sampling courses.

The participants wrapped the samples in ice cubes and thermal bags and froze them in their home freezer at −18 °C. This packaging allowed for transfer to the study site without thawing within 14 d for further storage at −80 °C. We collected a sample from each stool donor's first donation and immediately froze it at −80 °C.

### Bile acid analysis

The frozen stool samples were lyophilized and then homogenized by grinding them into a fine powder. They were weighed, and an internal synthesis standard was added before extraction. After deproteinization and alkalinization, the samples were loaded into solid-phase extraction C18 Chromabond Cartridges (Macherey-Nagel, Düren, Germany), and elution was performed with methanol. The extracted bile acids were separated by high-pressure liquid chromatography-tandem mass spectrometry (HPLC MS/MS). The bile acids were quantified, yielding concentrations of 28 different bile acids (Table 2) in nmol/g of lyophilized feces in each stool sample after calibration of the method with weighted mixtures and normalization relative to the internal bile acid standards. For quantitation, data were acquired using Analyst V.1.4.2 software. The preparation and analysis methods have formerly been described.^27^

**Table 2.** The 28 measured bile acids (Table adapted from Palmieri et al.^28^).

Primary bile acids		Secondary bile acids		Ursodeoxycholic acids	
CA TCA GCA CA-3S CDCA TCDCA GCDCA CDCA-3S HCA MCA		DCA TDCA GDCA DCA-3S LCA TLCA GLCA LCA-3S TLCA-3S GLCA-3S HDCA THDCA		UDCA TUDCA GUDCA UDCA-3S TUDCA-3S GUDCA-3S	
Abbreviations					
CA	Cholic acid		Pre-/suffixes:		
CDCA	Chenodeoxycholic acid		T		Tauro-conjugated
HCA	Hyocholic acid		G		glyco-conjugated
MCA	Muricholic acid		3S		sulfated on carbon 3
DCA	Deoxycholic acid				
LCA	Lithocholic acid				
HDCA	Hyodeoxycholic acid				
UDCA	Ursodeoxycholic acid				

### Analysis of known bile acid-transforming bacteria

We analyzed stool samples for the presence of some bacterial species involved in the transformation from primary bile acids into secondary bile acids, e.g., *C. scindens*, *C. hylemonae, C. hiranonis, C. leptum*, and *C. sordellii*^6^^-^^9^ , using 16S rDNA gene sequencing. We targeted the 16S rDNA gene with a modified version of the published universal prokaryotic primers 341F/806R. These primers were used to amplify the purified DNA from the samples with a short PCR setup. The following sequencing was performed using the Illumina MiSeq desktop sequencer and the 500-cycle MiSeq Reagent Kit V2 in a 2x250nt setup (Illumina Inc., San Diego, CA 29122, USA). We mapped the sequence data using the BION software, which is a k-mer-based approach for species-level annotation against the Ribosomal Database Project (RDP). The method has been published elsewhere.^29^ To verify the correct identification of the five selected bacterial species, consensus sequences from the created clusters were analyzed using the BLASTn tool against the NCBI GenBank database to confirm the species ID.^30^

Samples with low counts of total number of reads (<3000 reads) were considered unreliable and excluded from analyses. We calculated the relative abundance of the selected species in the samples as the number of reads of the species out of the total number of reads in percent (%).

### Statistical analyses

Continuous data was described with the median (interquartile range (IQR) and range), and categorical data with numbers and percentages/proportions. We compared continuous data with the Wilcoxon Rank Sum test in the case of comparing two groups. When comparing more than two groups, we used the Kruskal‒Wallis test followed by a pairwise Wilcoxon Rank Sum test to further explore a possible difference between groups.

A significance level of 0.05 was used in all analyses. All statistical analyses were performed in R version 4.5.0, while most graphic work was performed in GraphPad Prism version 10.2 (GraphPad Software, San Diego, CA, US).

### Ethical considerations

All stool donors and trial participants signed an informed consent form before participation, and the Regional Committee of Health Research Ethics in Region Zealand (SJ-478), the Danish Medicines Agency (EudraCT 2015-003062-82), and the Danish Data Protection Agency (REG-103-2015) approved the study. Furthermore, the randomized controlled trial was registered at ClinicalTrials.gov (NCT02774382, registered 2016-05-17). The study was performed in accordance with the Declaration of Helsinki II.

## Results

Twenty-two participants were included in the subgroup for bile acid analyses. The study population, the overall clinical cure rates, and the baseline characteristics of the subgroup participants are shown in Figure 1B and Table 3. Ten stool donors served as healthy controls (Figure 1B).

**Table 3.** Baseline characteristics of participants with recurrent *C. difficile* infection.

	All *n* = 21	RBT *n* = 8	FMT *n* = 7	Vancomycin *n* = 6
Age, years;				
median(IQR)	69(63–76)	68(58–71)	74 (70–79)	70 (59–78)
(range)	(34–86)	(36–79)	(64–86)	(34–84)
Sex ‐ Women; *n* (%)				
	17 (81%)	7 (88%)	5 (71%)	5 (83%)
Number of recurrences;				
median (IQR)	2 (1–2)	2 (1.8–2.3)	1 (1.0–1.5)	2 (2.0–2.0)
maximum	4	4	2	2
1. recurrence of CDI	8 (38%)	2 (25%)	5 (71%)	1 (17%)
≥2 recurrences of CDI	13 (62%)	6 (75%)	2 (29%)	5 (83%)
Charlson Comorbidity Index, CCI;				
median (IQR)	2 (0–3)	1.5 (0.8–2.3)	3.0 (0.0–4.0)	1.0 (0.0–2.8)
(range)	(0–5)	(0–4)	(0–5)	(0–5)
Hospital admission at inclusion;				
Hospital admission at inclusion; *n* (%)	1 (5%)	0	1 (14%)	0
BMI;				
median (IQR)	23.7 (21–27)	24.2 (22–29)	22.5 (21–26)	25.4 (22–27)
(range)	(18–37)	(19–37)	(20–29)	(18–27)
Toxin-profile; *n* (%)				
Toxin B	15 (71%)	7 (88%)	4 (57%)	4 (66%)
Binary toxin	6 (29%)	1 (12%)	3 (43%)	2 (33%)
CD027	0	0	0	0

RBT: rectal bacteriotherapy; FMT: fecal microbiota transplantation; IQR: interquartile range; CDI: *Clostridioides difficile* infection. Toxin-profile: according to PCR-test for *C. difficile* toxin genes. Toxin B: strain with the gene for toxin B; Binary toxin: strain with genes for both toxin B and binary; CD027: Strain with a deletion in the tcdC gene, presumptive PCR ribotype 027.

### Bile acid composition at baseline

**Figure 2.**
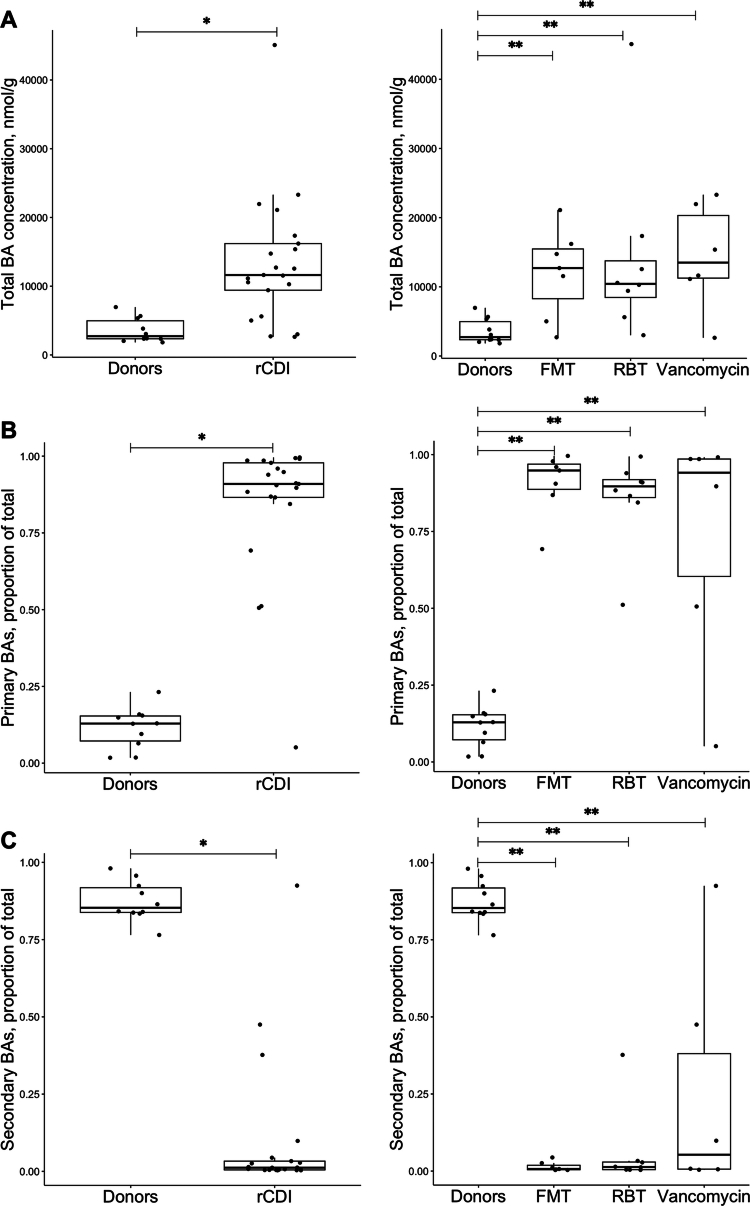
Comparisons of bile acid content in stool samples between healthy donors and participants with recurrent *C. difficile* infection at baseline (left) and comparisons between healthy donors and each treatment group at baseline (right). (A) Total concentration of bile acids (nmol/g dried feces); (B) Proportion of primary bile acids of the total amount of bile acids; (C) Proportion of secondary bile acids of the total amount of bile acids. *Wilcoxon Rank Sum test, *p* ≤ 0.0001; **Kruskal‒Wallis test followed by pairwise Wilcoxon Rank Sum test, *p* ≤ 0.01. BAs: bile acids; FMT: fecal microbiota transplantation; RBT: rectal bacteriotherapy.

**Figure 3.**
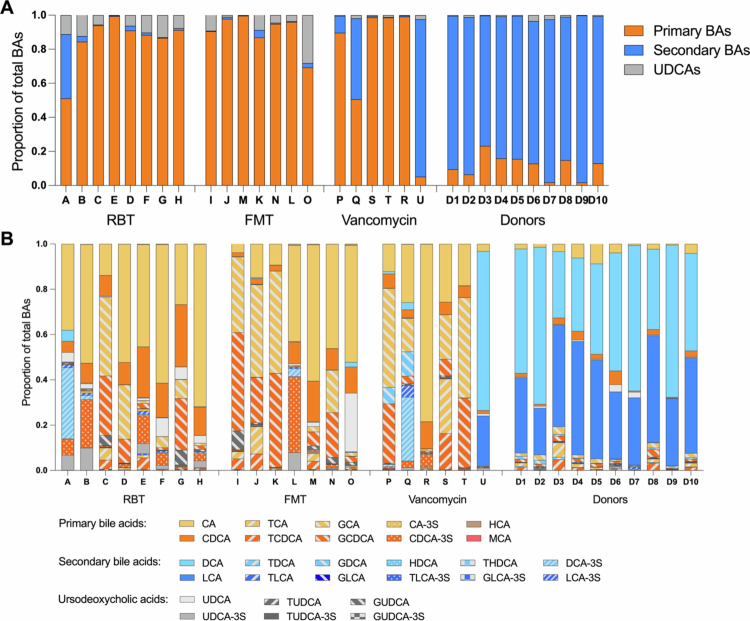
Bile acid composition in proportion of total content in stool samples from individuals (A-U) in each treatment group at baseline and in individual donors (D1–D10). (A) Divided into primary bile acids, secondary bile acids, and UDCAs in proportion to the total bile acid content. (B) Divided into 28 measured bile acid subtypes in proportion of total bile acid content. BA: bile acid; RBT: rectal bacteriotherapy; FMT: fecal microbiota transplantation; UDCA: ursodeoxycholic acid. Abbreviations for BA subtypes are shown in Table 2.

The content of bile acids in stool samples differed quantitatively and qualitatively between participants with recurrent *C. difficile* infection and healthy donors. Participants with recurrent *C. difficile* infection resulted in a significantly higher total concentration of bile acids, a higher proportion of primary bile acids, and a lower proportion of secondary bile acids (Figure 2). There were no differences among the three treatment groups at baseline. Thus, most participants had primary bile acids corresponding to more than 80% of the total content in their stool at baseline, while most donors had more than 80% secondary bile acids (Figure 3A). Notably, one participant in the vancomycin group (individual U) already had a donor-like bile acid composition at baseline.

We observed some individual variations in the subtypes of bile acids in samples from the participants with recurrent *C. difficile* infection. Nevertheless, the primary bile acids CA dominated (median proportion of total: 38% *versus* 3% in donors, *p* < 0.0001), while also CDCA (9% *versus* 2% in donors, *p* < 0.001) and the glyco-conjugated derivates of CA and CDCA were more abundant than they were in donor samples (Figure 3B and Supplementary Materials, Table S1).

### Bile acid composition in healthy donors

As expected in healthy individuals, the main secondary bile acids, DCA and LCA, dominated the composition for all donors (Figure 3B and Supplementary Material, Table S2).

The donors used for FMT in the subgroup (D1, D8, D9, and D10) did not differ from the entire donor population (Supplementary Material, Table S2b). Nonetheless, one donor, which was not used for participants in the subgroup, stood out. Donor D3 tended to have a higher proportion of primary bile acids and, vice versa, a lower proportion of secondary bile acids than the other donors. The primary bile acids carried by this donor were dominated by conjugated bile acids, mainly TCA (6.4% in D3 *vs.* 0.1%–1.6% in the other donors). TCA is known to be a potent germinant for *C. difficile*. Hence, in theory, donor D3 could be inappropriate for FMT for recurrent *C. difficile* infection. To explore this theory, we evaluated the clinical response after FMT with this donor. D3 was used only once in the randomized controlled trial (as infusion number three of three) but was used for eight off-protocol patients with recurrent *C. difficile* infection. Only three of these nine FMT recipients achieved a clinical cure (33%), i.e., lower than expected with an overall cure rate in the study of 56%–76% after FMT.^4^ Another donor, D8, also exhibited a rather high proportion of conjugated bile acids, yet this was due to higher levels of glyco- and tauro-conjugated CDCAs, which are possible inhibitors of *C. difficile*. The level of TCA in D8 was like the other donors and the rate of clinical cure seen after FMT (in the trial and off-protocol) with this donor was as expected (64%).

### Bile acid composition following treatments

After the three trial treatments for recurrent *C. difficile* infection, most participants had substantial shifts in the faecal content of bile acids, including a marked reduction in the total bile acid concentration (Supplementary Materials, Table S3), a decrease in the proportion of primary bile acids and, conversely, an increase in the proportion of secondary bile acids – all leading to a donor-like bile acid profile.

**Figure 4.**
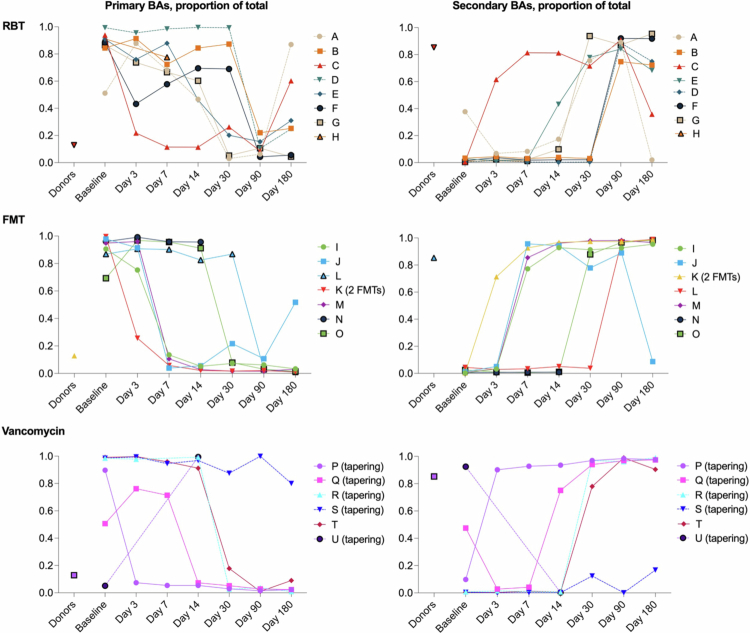
The proportions of primary bile acids (left) and secondary bile acids (right) of the total content of bile acids over time after treatment on an individual level (A-U) for each treatment group. Each individual is depicted with a unique color. Dashed lines: Participants experiencing failure of treatment. Solid lines: Participants with a clinical cure at 90-d follow-up. The median level for healthy donors is shown in each plot for comparison. BAs: bile acids; RBT: rectal bacteriotherapy; FMT: fecal microbiota transplantation.

The shifting bile acid compositions in individuals are shown for each treatment group in Figure 4.

In the rectal bacteriotherapy group, all participants recovered to a normal composition at some time after treatment, yet only one participant (C) shifted immediately after, while the rest shifted before day 30 or 90 with a tendency to return to baseline levels at the end of the study period (Figure 4, top). Notably, two of the participants (A and D) had a new recurrence of *C. difficile* infection after rectal bacteriotherapy (dashed lines in Figure 4) and were treated with vancomycin followed by FMT off-protocol between day 30 and day 90.

After FMT, six of seven participants shifted from a dominance of primary bile acids to a dominance of secondary bile acids – four already before day 7 and two before either day 30 or 90. The last participant did not deliver samples after day 14, when no shift had occurred (individual *N*).

Notably, one individual (K) received two FMTs with two different donors 6 d apart according to the protocol due to an insufficient clinical response after the first. For this individual, the depicted day 3 is 3 d after the second FMT. An extra sample from the two FMTs showed that the bile acid composition was unchanged before the second FMT (Supplementary Materials, Figure S1). All participants in the FMT group successfully recovered with no further recurrences (Figure 4, middle).

Four of the six participants in the vancomycin-group, recovered to donor-like levels of bile acids – one already during/just after vancomycin intake (*P*), one before day 14, and two before day 30. Of the remaining two, one did not re-establish normal composition during the study period (S), the other (U) experienced a rise in primary bile acids after vancomycin and did not produce samples after day 14 (Figure 4, bottom).

One individual (T) only received a 14-d course of vancomycin, while the others (P, Q, R, S, and U) all received 14 d of full-dose vancomycin followed by an additional 5-week tapering course because they had experienced more than one recurrence. Note that sampling began after the final dose of the tapering course.

Three participants in the vancomycin group had further recurrences (R, S, and U, dashed lines in Figure 4), two of them already during tapering of vancomycin (i.e., before day 1), leading to later FMT off the protocol, which could have affected the measured bile acid contents afterwards. Individual U had a donor-like bile acid profile at baseline but seemed refractory to treatment. Diarrhea increased during tapering, after which the vancomycin dose was increased, followed by five FMTs before the patient became diarrhea free. Unfortunately, she failed to deliver any samples after day 14 (before FMTs).

**Figure 5.**
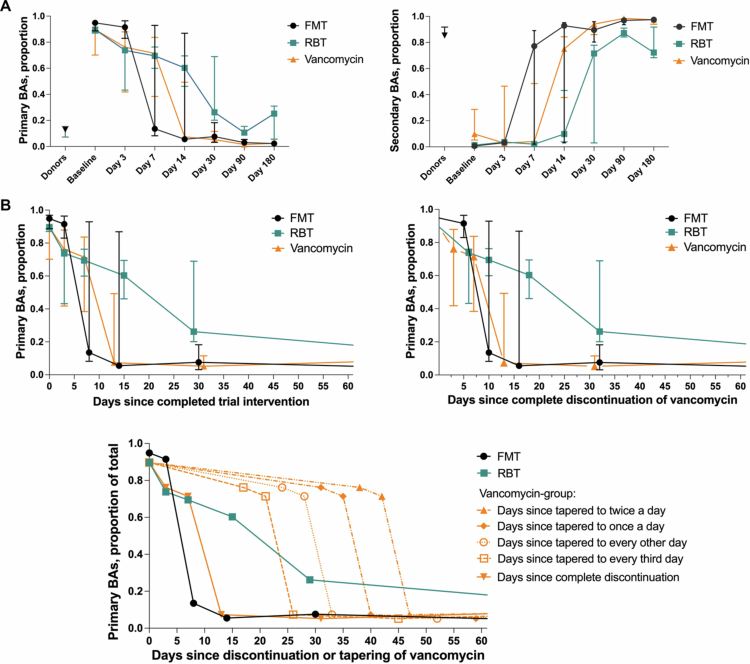
(A) Median proportions of primary bile acids (left) and secondary bile acids (right) after each treatment regimen for participants with treatment success, i.e., clinical cure at 90 d. To compare the treatments, we excluded the sampling times that were inconsistent between groups to obtain uniform time courses, i.e., excluding the samples just before rectal bacteriotherapy/FMT (day 0) and the day after the last vancomycin-intake in the vancomycin-group (day 1). Points = median, error bars = interquartile range. The median proportions in stool samples from healthy donors are shown for comparison. BAs: bile acids; RBT: rectal bacteriotherapy; FMT: fecal microbiota transplantation. B. Left: Median proportions of primary bile acids according to the absolute number of days since completion of each treatment regimen for participants with treatment success, i.e., clinical cure at 90 d. Right: Median proportions of primary bile acids according to the absolute number of days since complete discontinuation of vancomycin for participants with treatment success, i.e., clinical cure at 90 d. Below: Median proportions of primary bile acids according to the absolute number of days since discontinuation of vancomycin in the FMT- and RBT-group, compared with the median proportions of primary bile acids according to the number of days since complete discontinuation of vancomycin in the vancomycin-group (*n* = 3) and compared with the different steps of tapering of the dose for participants receiving tapering in the vancomycin-group (*n* = 2). As above, only the participants with treatment success, i.e., clinical cure at 90 d, are included here. Note that the graphs’ x-axes are different from those in Figure 5A, where the x-axes are trial visits, while 5B is numerical. Further, the axes here end at 60 d to improve the visualization of the most relevant time period.

The median changes in bile acids after the three different treatments are shown in Figure 5A. To compare the treatments, we excluded the sampling times that were inconsistent between groups. In addition, individuals failing allocated treatment were excluded in this comparison, since they received other off-protocol treatment in the study period.

The recovery of bile acids towards low proportions of primary bile acids and high proportions of secondary bile acids seemed to occur the fastest in the FMT group, followed by the vancomycin group, and the rectal bacteriotherapy, which recovered somewhat later (Figure 5A). However, we only had a few participants in each treatment group (*n* = 6–8), especially when we were restricted to participants cured after the trial intervention (*n* = 3–7). Furthermore, there was a high inter-individual variation in each group. Hence, we did not attempt to perform statistical comparisons between the treatment groups.

As described, the study design led to inherent time differences in the treatment courses between groups, especially considering the timing of discontinuing vancomycin. It is important to notice that day 3 after treatments correspond to respectively 5 d after discontinuing vancomycin in the FMT group (yet 11 d for the one individual, K, where FMT was repeated), 7 d in the bacteriotherapy group and 3 d after complete discontinuation in the vancomycin-group, where 2 of 3 participants with treatment success received a tapered dose for 5 weeks before discontinuation (Figure 1). To account for these differences when interpreting the results, we also compared the development of bile acids in the three groups according to the absolute number of days since complete discontinuation of vancomycin and to the number of days since different steps of tapering for the participants in the vancomycin-group, since a slow partial recovery of the microbiome and the bile acid composition would be expected during tapering (Figure 5B). Here, the observed faster re-establishment of bile acids after FMT compared with vancomycin was less apparent when looking at the days since the complete discontinuation of vancomycin, with only a few days between the groups. In contrast, the difference was much more apparent when considering the tapering of the vancomycin dose.

We observed substantial inter-individual variations concerning the conjugation status and the most abundant subtypes of bile acids before and after treatments, yet some overall patterns were observed.

Participants with recurrent *C. difficile* infection tended to have higher proportions of conjugated bile acids at baseline than healthy donors (median proportion of total: 30% *versus* 6%, *p* = 0.09). In the bacteriotherapy- and FMT-groups, we observed an increase in conjugated bile acids after vancomycin pre-treatment with a secondary rapid decrease just after rectal bacteriotherapy and FMT to very low levels (Supplementary Materials, Figure S2, left), especially due to decreases in GCA and GCDCA. We observed a simultaneous extensive rise in the deconjugated CA and less dramatic increase in CDCA after rectal bacteriotherapy and FMT. After FMT, this was immediately followed by increases in LCA and especially DCA, while the proportion of LCA and DCA increased later in the bacteriotherapy group (Figures S3–S5). The pattern was less clear for participants in the vancomycin group, yet with an overall decrease in the proportion of conjugated bile acids after treatment ended.

### Presence of bile acid-transforming bacteria

**Figure 6.**
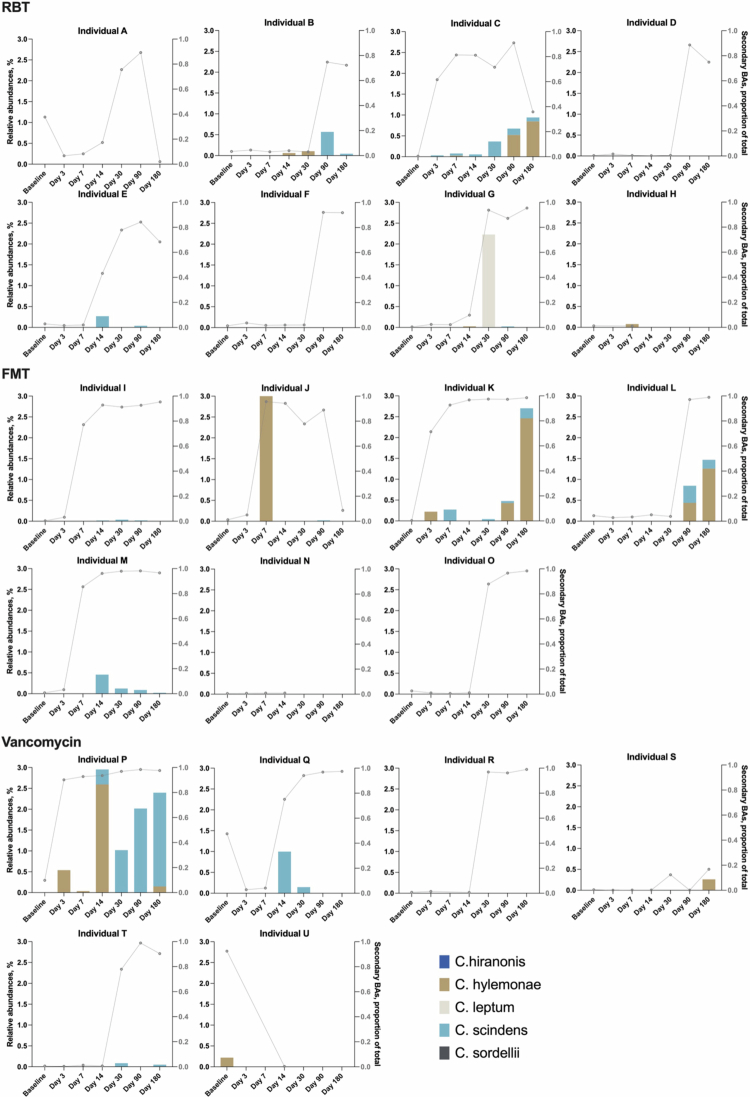
Relative abundances of *C. scindens*, *C. hylemonae, C. hiranonis, C. leptum*, and *C. sordellii* in percent (left Y-axis) over time before and after treatment on an individual level (A-U) for each treatment group are shown in bars. Each analyzed *Clostridium* species is shown in a different colours. For comparison, the concomitant proportions of secondary bile acids of the total content of bile acids (right y-axis in gray) are shown in connected dots in gray. Note that the left Y-axis is different for individual J to improve visualization. Note that we excluded donor D9, the baseline sample from individuals Q and T, the day 3 sample from individual M, and the day 90 sample from individual F. We further excluded days 7, 14, and 180 from individual R, the baseline sample, and days 7 and 30 from individual L, and six samples from individual O (all except the baseline sample). BAs: bile acids; RBT: rectal bacteriotherapy; FMT: fecal microbiota transplantation.

The relative abundances of the selected bacterial species (e.g., *C. scindens*, *C. hylemonae, C. hiranonis, C. leptum*, and *C. sordellii*) together with the proportion of secondary bile acids over the sample time points is visualized in Figure 6 for each individual (A-U). Quantitative abundance data are provided in the Supplementary Materials, Table S4. Owing to an unreliable low number of reads, several samples were excluded from analysis, including one donor (D9) and one sample from 4 individuals (F, M, Q, and T), three samples from 2 individuals (R and L), and 6 samples from one individual (O).

Surprisingly, we could not detect the presence of the selected *Clostridium* species in any of the donors. However, all donors consistently had reads indicating another *Clostridium* species, which unfortunately could not be further categorized (data not shown).

At baseline, samples from two of the participants contained some of the species investigated. The sample from individual *P* included *C. hiranonis*, though in very low amounts (relative abundance of 0.003%). Furthermore, Individual U's sample contained *C. hylemonae*. Interestingly, this was the same participant that had a donor-like bile acid profile at baseline.

In approximately half of the participants (10/21, i.e., individuals B, C, E, G, J, K, L, P, Q, and T), we observed a timely association between new reads of one or more of the tested bile acid-transforming species and a rise in the proportion of secondary bile acids (Figure 6). However, we also observed that some participants showed a clear increase in secondary bile acids without the appearance of any of the species (individuals A, D, F, I, O, and R). However, for individuals F, O and partly R, samples at the relevant time points were with very low counts in general and thus excluded (none of which had any reads of the relevant species).

We mostly detected *C. scindens* and *C. hylemonae* in the samples, except for individual G, where a temporary presence of *C. leptum* was observed. Furthermore, as described, one individual (P) had *C. hiraronis* at baseline, while this species was also detected at a low relative abundance (0.004%) in individual B at day 3. *C. sordellii* was detected in only one individual (S) at day 3, with a relative abundance of only 0.008%.

## Discussion

We performed a sub-study nested in a randomized controlled trial comparing the effect of FMT *versus* a standardized bacterial mixture (rectal bacteriotherapy) *versus* oral vancomycin for patients with recurrent *C. difficile* infection to explore whether re-establishment of a normal colonic bile acid composition^6^^-^^8^ is a mechanism of action in FMT. This study confirmed that patients with recurrent *C. difficile* infection have an abnormal bile acid profile distinct from that of healthy donors. The results indicated that FMT may re-establish a normal bile acid profile faster than both vancomycin and rectal bacteriotherapy. Furthermore, the results also indicated that the bile acid profile in donor stool could be a potential biomarker for donor selection.

As expected, participants with recurrent *C. difficile* infection had a fecal bile acid profile dramatically distinct from that of healthy donors at baseline, with a high dominance of primary bile acids, especially CA, and, to a lesser extent, CDCA, GCA, and GCDCA. These findings are consistent with those of former studies,^14-16^^,^^18^^,^^20^ while some studies also reported high abundances of TCA and TCDCA in patients with recurrent *C. difficile* infection.^17^^,^^19^^,^^31^ In *in vitro* studies on different bile acids ´ impact on *C. difficile*, TCA, CA, and GCA promoted the germination of spores into vegetative bacteria, whereas CDCA inhibited germination.^6-8^^,^^10^^,^^11^ Hence, the high proportions of CA and GCA found here, and by others, support the theory that patients with recurrent *C. difficile* infection harbor an environment friendly to *C. difficile.* Nevertheless, we also observed a somewhat high proportion of CDCA in these patients, though much lower than CA. Thus, the possible inhibitory effect may have been overridden. Surprisingly, TCA was not abundant in our population, despite its known positive effect on *C. difficile* germination. Albeit, it is likely that the inconsistencies found between *in vitro* studies and studies on patients with recurrent *C. difficile* infection can be explained by bile acids being found at other concentrations and acting differently *in vivo* than *in vitro*, since interactions with the complex gut ecosystem and between bile acids may modify the effect *in vivo.*^11^

One patient with recurrent *C. difficile* infection (individual U) stood out with a donor-like bile acid profile dominated by secondary bile acids and the presence of *C. hylemonae* at baseline. This should, in theory, protect against *C. difficile*. Nevertheless, this participant seemed treatment-refractory, indicating that other factors influence susceptibility to *C. difficile*. However, it is also likely that she was misdiagnosed and had diarrhea of another cause with concomitant *C. difficile* carriage. She was young (34 y) and treatment-refractory – both factors that have been linked to misdiagnosis of *C. difficile* infection.^32^

At baseline, we observed a total concentration of bile acids was approximately three times higher in stool of patients with recurrent *C. difficile* infection than from donors. These high concentrations of predominantly primary bile acids may in itself affect colonic transit time and secretion, leading to diarrhea independently of *C. difficile.* This has been shown in both patients with bile acid diarrhea, in some patients with diarrhea-predominant irritable bowel syndrome (IBS-D), and in healthy controls exposed to orally administered primary bile acids.^33^^-^^37^ In contrast to our findings, one former study reported an increased total concentration of bile acids after FMT.^16^ On the other hand, a study reported an increase in fibroblast growth factor 19 (FGF19), which inhibits bile acid synthesis, after FMT.^18^ Thus, a low level before FMT would result in high bile acid synthesis, which could also be the case in our study. This impairment of the negative feedback mechanism for bile acid synthesis could be caused by inflammation or *C. difficile* in itself.^18^ Moreover, a reduced transit time due to diarrhea can decrease the reabsorption of bile acids, reduce the microbial transformation of bile acids through decreased exposition, and perhaps affect the FGF19 pathway, leading to increased synthesis, as observed in IBS-D.^36^^,^^38^ Hence, we need a better understanding of these possible mechanisms in recurrent *C. difficile* infection.

The analysis of the bile acid profile in donors suggested a potential biomarker for the clinical efficacy of donor stool, which deserves further investigation. The bile acid composition of the donors was dominated by the main secondary bile acids, as expected in healthy individuals^27^ and as previously reported,^15^^,^^17^^,^^20^ yet one donor had a rather high proportion of TCA, and the clinical results indicated that material from this donor was less effective in FMT. We are unaware of the cause of this divergent bile acid profile, since the donor did not stand out on any of the screening questions or tests before and after donations. More data is needed to further investigate whether bile acid profiles in donors can be used to predict the effect of FMT and potentially aid in donor selection.

After treatment, most participants in our trial shifted towards a donor-like bile acid profile from a high dominance of primary bile acids to a high dominance of secondary bile acids during the follow-up period, regardless of the allocated treatment. However, this shift seemed to occur somewhat faster after FMT than after vancomycin monotherapy or rectal bacteriotherapy – both treatments where the clinical recovery rates were lower than for FMT. This could indicate that FMT may reduce the time interval during which the colonic environment favours *C. difficile* re-colonisation. Reducing this window of high susceptibility to *C. difficile* may reduce the risk of further recurrences. In support of this theory, an increase in microbiome richness and Shannon diversity (unpublished data) occurred in the FMT group simultaneously with changes in bile acid composition and lasted for the entire follow-up period. No similar increase in richness or diversity was found after treatment with rectal bacteriotherapy or vancomycin monotherapy.

Unfortunately, our modest sample size and the study design – with differences in the timing of vancomycin discontinuation between groups and the tapering of the dose for some in the vancomycin group – prevent us from drawing any conclusions.

Correcting for the time since discontinuation of vancomycin (Figure 5B) indicated that the difference in bile acid recovery between participants treated with FMT *versus* vancomycin was only a few days. However, this could be due to the long tapering regimen for participants with multiple recurrences in the vancomycin-group, since this might lead to a gradual re-establishment of the microbiome and the bile acid composition during the treatment. On the other hand, rectal bacteriotherapy did not seem to improve the re-establishment of the bile acid composition and might even delay the process.

Since all former studies evaluating bile acid composition after FMT for *C. difficile* infections were single-group studies, they were unable to distinguish changes observed after FMT from changes possibly induced by discontinuation of vancomycin.^15^^-^^20^ In our vancomycin group, the rate of recovery of the bile acid composition seemed highly variable among the three participants experiencing a clinical cure. However, for all of them, the shift occurred within 30 d, indicating a natural recovery of bile acid metabolism after *C. difficile* infection over time.

Only a few former studies included longitudinal data after FMT,^17^^,^^18^^,^^20^^,^^21^ in general, former studies did not evaluate changes early after FMT,^15^^,^^16^^,^^18^^,^^21^ and moreover, sampling times were quite heterogenic,^15^^-^^21^ which makes it hard to compare results.

In an *in vitro* clindamycin-exposed model for *C. difficile* infection, it was observed that the levels of bile acids normalized shortly after discontinuing clindamycin and, importantly, before adding donor stool.^39^ Nevertheless, the recovered bile acids were unable to inhibit *C. difficile* in this model. Although this study was *in vitro* andthe findings are not necessarily applicable to patients with recurrent *C. difficile* infection, these observations add to the complexity of this area.

Nevertheless, the significance of the bile acid profile in the recurrence of *C. difficile* infection was also supported in a prospective longitudinal study examining the content of bile acids in stool samples from patients after treatment of a first *C. difficile* infection.^40^ Patients with no recurrence showed recovery of their secondary bile acids, while presence of certain primary bile acids seemed to predict recurrence within the first 9 d after treatment.

In our study, we observed an increase in conjugated bile acids after vancomycin (pre-)treatment. This might be due to the depletion of microbiota able to produce bile salt hydrolases for deconjugation. The rapid shift from conjugated to deconjugated bile acids after FMT and rectal bacteriotherapy could indicate that both donor stool and the bacterial mixture add bile salt hydrolases directly or by adding bacteria producing these hydrolases. In the FMT group, the subsequent shift towards a dominance of secondary bile acids could be induced by transfer of bacteria or enzymes involved in 7α-dehydroxylation – a process that needs prior deconjugation to occur.^41^

Many of the former studies did not report the conjugation status of bile acids.^15^^,^^17^^,^^18^^,^^20^^,^^21^ One former study demonstrated the restoration of bile salt hydrolase activity after FMT and hypothesized that this step in bile acid metabolism can be a key mechanism of action in FMT.^19^ These findings indicated that bile salt hydrolase activity could be sufficient in suppressing *C. difficile* germination and possible recurrence of *C. difficile* infection *in vitro* and in a mouse model. Our study does not clearly answer the question of whether bile salt hydrolase or 7α-dehydroxylation activity is the key factor, yet the observations in the bacteriotherapy-treated participants suggest that both properties are necessary. The bacterial mixture seemed to add bile salt hydrolase activity, yet, the transformation to secondary bile acids seemed to occur late, and importantly, we observed a disappointing clinical effect of rectal bacteriotherapy in the randomized controlled trial.^4^

Several different gut bacteria produce bile salt hydrolase for deconjugation, while 7α-dehydroxylation is believed to be reserved for only a few species.^6^^-^^9^ We performed 16S rDNA gene sequencing to analyze the abundance of these species and partly observed a timely association between the increase in the proportion of secondary bile acids and the appearance of these species, especially *C. scindens* and *C. hylemonae*. However, this pattern was not consistent, and importantly, and opposed to what we expected, we could not identify any of the selected species in the donor samples. This raises several questions for further investigation. We must assume that the donors carry the ability to conduct 7α-dehydroxylation and transform primary bile acids to secondary bile acids as healthy individuals.^6^^-^^8^ Thus, one possibility is that there are other species able to conduct this process than the ones analyzed. Further investigation into this topic is needed. Another possibility is that the shift in the bile acid composition is induced by direct transfer of enzymes rather than bacteria producing these enzymes, yet this is only possible for the patients receiving FMT. Unfortunately, we did not evaluate bile salt hydrolase or 7α-dehydroxylation activity directly.

Moreover, the appearance of the selected bacterial species after treatments does not seem to be due to direct engraftment of the donor content. Thus, the source of these species is unknown but could be due to a general shift in the microbiome, possibly inducing an increase in the abundance of these species from existing yet undetectable low levels at baseline. Further analyses of the change in the microbiome after the three treatments are needed.

Despite these unanswered questions, it is possible that adding *C. scindens* or *C. hylemonae* to the bacterial mixture could improve the clinical effect in treating recurrent *C. difficile* infection. In support of this theory, one study treated *C. difficile*-infected mice with *C.* *scindens-*strains and reported an increased *C. difficile* resistance and survival.^42^

Notably, we observed someparticipants in all three treatment groups experiencing clinical cure, despite a late recovery of the bile acid composition, . Thus, this proposed mechanism of action does not explain the effect alone. However, it may be a piece of a multifactorial complex of mechanisms. Moreover, our data do not reveal whether the recovery of bile acid homeostasis is important in itself or a proxy for the recovery of the gut microbiota and its metabolic pathways.

Our study has some clear limitations, including a small sample size, the lack of full characterization of the microbiome (e.g., with shotgun metagenomic sequencing), and a study design with differences in the timing of treatments and sampling between groups. All of these limitations hamper firm conclusions. Furthermore, we selected the subgroup from the randomized controlled trial in a non-random manner for practical reasons, which could introduce selection bias and leave the three groups with some basic differences. For example, we observed that more participants in the FMT group had a first recurrence than in the other two groups. However, to our knowledge, it is unknown if bile acid metabolism differs between patients with a first or second recurrence, and it seems unlikely that potential differences are substantial.

In conclusion, we confirmed that recurrent *C. difficile* infection is associated with an imbalance in the colonic bile acid profile, which may be crucial for susceptibility to *C. difficile.* It appears that FMT could re-establish a normal bile acid composition faster than observed after vancomycin and the investigated rectal bacteriotherapy. If this is the case, FMT may (partly) work by introducing or stimulating either bacteria or metabolites involved in bile acid homeostasis, thereby reducing the timeframe where the patient is vulnerable to re-colonisation of *C. difficile* and recurrences. It is possible that standardized bacterial mixtures developed as alternatives to donor stool may benefit from bacteria involved in bile acid metabolism. Finally, the bile acid profile in donor stool could have the potential to predict the effect of FMT and can perhaps serve as a biomarker for the selection of optimal donors.

## Supplementary Material

Supplementary Materials_gut microbes_vers3.docx Supplementary Materials_gut microbes_vers3.docx

## Data Availability

All datasets generated and analyzed during the present study are included in the published article and its supplementary information files.

## Abbreviations

FMT: Fecal microbiota transplantation

### Abbreviations for bile acid subtypes

CA: Cholic acid
CDCA: Chenodeoxycholic acid
HCA: Hyocholic acid
MCA: Muricholic acid
DCA: Deoxycholic acid
LCA: Lithocholic acid
HDCA: Hyodeoxycholic acid
UDCA: Ursodeoxycholic acid

### Pre-/suffixes on bile acid subtypes

T: Tauro-conjugated
G: Glyco-conjugated
3S: Sulfated on carbon 3
