# Supplementary material for: Re-establishing bile acid composition after treatment of recurrent Clostridioides difficile infection with fecal microbiota transplantation compared with oral vancomycin or a 12-strain bacterial mixture
Source: Gut Microbes. 2026 Apr 17;18(1):2658915. doi: 10.1080/19490976.2026.2658915 (PMC13094210; doi:10.1080/19490976.2026.2658915)
Supplement: Supplementary Materials_gut microbes_vers3.docx [file KGMI_A_2658915_SM4119.docx]

**Supplementary materials to**

**Re-establishing bile acid composition after treatment of recurrent *Clostridioides difficile* infection with faecal microbiota transplantation compared with oral vancomycin or a 12-strain bacterial mixture**

Anne Abildtrup Rode^1,2^, Henri Duboc^3,4^, Antonin Lamazière^5^, Dominique Rainteau^5^**^‡^**, Lydie Humbert^5^**^‡^**, Emilie Gauliard^5^, Mahtab Chehri^2,6^, Andreas Munk Petersen^2,6,7^, Morten Helms^2,8^, Kristian Schønning^2,7†^, Peter Bytzer^1,2^, Jørgen Engberg^9^

^1^ Department of Medicine, Zealand University Hospital, Koege, Denmark

^2^ Department of Clinical Medicine, University of Copenhagen, Denmark

^3^ Université Paris Cité, CRI Inserm UMRS 1149, équipe PIMS, F-75018 Paris, France

^4^AP-HP, Hopital Louis Mourier, DMU ESPRIT, Department of Gastroenterology, F-92700 Colombes, France

^5^ Sorbonne Université, Inserm, Centre de Recherche Saint-Antoine, CRSA, AP-HP, Hôpital Saint Antoine, Clinical Metabolomics Department F-75012 Paris, France

^6^ Department of Gastroenterology, Hvidovre University Hospital, Denmark

^7^ Department of Clinical Microbiology, Hvidovre University Hospital, Denmark

^8^ Department of Infectious Diseases, Hvidovre University Hospital, Denmark

^9^ Department of Clinical Microbiology, Zealand University Hospital, Slagelse, Denmark

† Current workplace: Department of Clinical Microbiology, Rigshospitalet, Copenhagen University Hospital, Denmark

‡ Now retired

Correspondence should be addressed to Anne A. Rode, [aala@regionsjaelland.dk](mailto:aala@regionsjaelland.dk)

| **Table S1 - Bile acid subtypes as percentage of the total bile acid content measured at baseline for healthy donors and rCDI-patients in total and according to treatment-group,** *median % (IQR)* | | | | | |
| --- | --- | --- | --- | --- | --- |
|  | **Donors** | **rCDI (all)** | **FMT** | **RBT** | **Vancomycin** |
| *Primary BAs* | | | | | |
| **CA** | 2.8 (1.6–4.0) | **38.1 (14.8–52.1)** | **42.4 (12.0–48.8)** | **48.6 (35.2–54.5)** | **21.9 (13.7–25.6)** |
| **GCA** | 1.7 (0.9–2.2) | **8.3 (1.1–33.3)** | **18.9 (2.0–37.1)** | 3.1 (0.9–12.2) | **17.2 (4.4–37.7)** |
| TCA | 1.2 (0.4–1.6) | 0.4 (0.1–1.3) | 0.5 (0.4–3.3) | 0.3 (0.2–0.9) | 0.2 (0.1–0.4) |
| CA-3S | 0 | 0 (0–0.2) | 0 | 0.1 (0–0.4) | 0 (0–0.2) |
| **CDCA** | 2.3 (1.1–2.8) | **9.2 (4.9–11.9)** | **9.3 (2.5–10.6)** | **11.2 (9.1–17.0)** | **5.3 (3.9–6.1)** |
| **GCDCA** | 1.3 (0.8–1.5) | **6.2 (1.0–22.8)** | **19.8 (4.4–30.7)** | 1.9 (0.9–13.6) | **5.4 (1.3–21.5)** |
| TCDCA | 1.0 (0.3–1.4) | 0.6 (0.1–3.6) | 0.8 (0.4–4.2) | 0.6 (0.3–1.6) | 0.2 (0.1–0.5) |
| CDCA-3S | 0.1 (0–0.1) | 0.8 (0.4–5.2) | 0.5 (0..2–1.1) | 4.2 (1.3–8.5) | 0.7 (0.4–2.4) |
| HCA | 0 | 0.1 (0–0.2) | 0.1 (0–0.4) | 0.1 (0–0.2) | 0 (0–0.1) |
| MCA | 0 | 0 | 0 | 0 | 0 |
| *Secondary BAs* | | | | | |
| **DCA** | **47.6 (36.6–61.9)** | 0.1 (0.1–0.4) | 0.1 (0–0.4) | 0.1 (0.1–0.1) | 0.6 (0.1–2.6) |
| GDCA | 0.7 (0.4–0.8) | 0 (0–0.1) | 0 (0–0.1) | 0 | 0 (0–5.5) |
| TDCA | 0.3 ( 0.2–0.4) | 0 | 0 | 0 | 0 |
| DCA-3S | 0.1 (0.1–0.2) | 0.2 (0.1–0.6) | 0.1 (0–0.3) | 0.5 (0.1–0.9) | 0.2 (0–0.9) |
| **LCA** | **37.7 (29.7–44.6)** | 0.1 (0–0.1) | 0 (0–0.2) | 0.1 (0–0.1) | 0.1 (0–0.1) |
| GLCA | 0 | 0 | 0 | 0 | 0 |
| TLCA | 0 | 0 | 0 | 0 | 0 |
| LCA-3S | 0.2 (0.1–0.4) | 0.1 (0.1–0.6) | 0.1 (0–0.2) | 0.5 (0.1–1.1) | 0.1 (0.1–0.2) |
| GLCA-3S | 0 | 0.1 (0–0.1) | 0.1 ( 0–0.1) | 0 (0–0.1) | 0.2 (0–0.2) |
| TLCA-3S | 0 (0–0.2) | 0 (0–0.1) | 0 (0–0.1) | 0 | 0 (0–0.1) |
| HDCA | 0 | 0 (0–0.1) | 0 | 0.1 (0–0.2) | 0 |
| THDCA | 0 | 0 | 0 | 0 | 0 |
| *UDCAs* | | | | | |
| **UDCA** | 0.4 (0.4–0.8) | 0.3 (0.1–2.2) | 0.3 (0.1–1.3) | 2.8 (0.9–4.5) | 0.1 (0–0.2) |
| GUDCA | 0.1 (0–0.1) | 0.4 (0.2–1.3) | 1.3 (0.4–2.1) | 0.2 (0.2–1.8) | 0.3 (0.2–0.5) |
| TUDCA | 0 | 0 (0–0.4) | 0.2 (0–0.5) | 0.2 (0–0.4) | 0 |
| UDCA-3S | 0 (0–0.1) | 0.5 (0.1–1.8) | 0.4 (0.1–0.5) | 2.4 (0.2–5.1) | 0.3 (0.1–1.0) |
| GUDCA-3S | 0 | 0 | 0 | 0 | 0 |
| TUDCA-3S | 0 | 0 | 0 | 0 | 0 |

| **Table S2 - Bile acid subtypes measured at baseline for individual donors as percent** **of the total bile acid content, %** | | | | | | | | | | |
| --- | --- | --- | --- | --- | --- | --- | --- | --- | --- | --- |
|  | **D1** | **D2** | **D3** | **D4** | **D5** | **D6** | **D7** | **D8** | **D9** | **D10** |
| *Primary BAs* | | | | | | | | | | |
| **CA** | 2.2 | 1.4 | 3.3 | 6.2 | 8.7 | 3.9 | 0.6 | 2.2 | 0.4 | 4.1 |
| GCA | 1.5 | 2.4 | 3.3 | 1.4 | 2.0 | 0.7 | 0.2 | 2.3 | 0.2 | 2.0 |
| TCA | 1.5 | 0.3 | **6.4** | 1.6 | 0.9 | 0.6 | 0.1 | 1.5 | 0.2 | 1.6 |
| CA-3S | 0 | 0 | 0 | 0 | 0 | 0 | 0 | 0 | 0 | 0 |
| **CDCA** | 1.6 | 0.9 | 2.8 | 3.8 | 2.1 | 6.1 | 0.5 | 2.3 | 0.4 | 2.7 |
| GCDCA | 1.4 | 1.1 | 2.3 | 1.5 | 1.4 | 0.8 | 0.3 | **3.3** | 0.4 | 1.0 |
| TCDCA | 1.4 | 0.2 | 4.9 | 1.2 | 0.5 | 0.7 | 0.1 | **3.2** | 0.2 | 1.4 |
| CDCA-3S | 0 | 0.1 | 0.2 | 0.1 | 0 | 0.1 | 0 | 0.1 | 0 | 0.2 |
| HCA | 0 | 0 | 0 | 0 | 0 | 0 | 0 | 0 | 0 | 0 |
| MCA | 0 | 0 | 0 | 0 | 0 | 0 | 0 | 0 | 0 | 0 |
| **Total** | 9.5 | 6.5 | **23.2** | 15.9 | 15.5 | 12.8 | 1.8 | 14.9 | 1.8 | 13.0 |
| **Conjugated** | 5.7 | 4.1 | **16.9** | 5.8 | 4.7 | 2.8 | 0.7 | **10.2** | 0.9 | 6.0 |
| Glyco-conjugated | 2.9 | 3.6 | 5.6 | 2.9 | 3.3 | 1.5 | 0.5 | 5.5 | 0.6 | 2.9 |
| Tauro-conjugated | 2.9 | 0.5 | **11.3** | 2.9 | 1.3 | 1.3 | 0.2 | 4.7 | 0.3 | 3.0 |
| **Sulfated** | 0 | 0.1 | 0.2 | 0.1 | 0 | 0.1 | 0 | 0.1 | 0 | 0.2 |
| *Secondary BAs* | | | | | | | | | | |
| **DCA** | 54.9 | 69.3 | 29.3 | 32.3 | 39.9 | 52.1 | 64.2 | 35.5 | 67.2 | 43.1 |
| GDCA | 1.3 | 1.7 | 0.7 | 0.3 | 0.2 | 0.3 | 0.4 | 0.7 | 0.9 | 0.6 |
| TDCA | 0.5 | 0.2 | 1.0 | 0.1 | 0.1 | 0.4 | 0.1 | 0.4 | 0.2 | 0.3 |
| DCA-3S | 0.1 | 0.3 | 0.1 | 0.2 | 0.1 | 0.1 | 0.4 | 0.1 | 0.1 | 0.1 |
| **LCA** | 33.2 | 20.6 | 45.0 | 50.1 | 43.6 | 29.7 | 29.7 | 47.3 | 29.5 | 42.1 |
| GLCA | 0 | 0 | 0 | 0 | 0 | 0 | 0 | 0 | 0 | 0 |
| TLCA | 0 | 0 | 0 | 0 | 0 | 0.1 | 0 | 0.1 | 0 | 0 |
| LCA-3S | 0.1 | 0.3 | 0.1 | 0.4 | 0 | 0.4 | 0.8 | 0.1 | 0 | 0.2 |
| GLCA-3S | 0 | 0 | 0 | 0 | 0 | 0.3 | 0 | 0 | 0.1 | 0 |
| TLCA-3S | 0 | 0 | 0.3 | 0 | 0 | 0.2 | 0 | 0 | 0 | 0 |
| HDCA | 0 | 0 | 0 | 0 | 0 | 0 | 0 | 0 | 0 | 0 |
| THDCA | 0 | 0 | 0 | 0 | 0 | 0 | 0 | 0 | 0 | 0 |
| **Total** | 90 | 92.4 | 76.5 | 83.4 | 84.0 | 83.7 | 95.7 | 84.2 | 98.0 | 86.4 |
| **Conjugated** | 1.8 | 1.9 | 2.0 | 0.4 | 0.3 | 1.4 | 0.5 | 1.2 | 1.2 | 0.9 |
| Glyco-conjugated | 1.3 | 1.7 | 0.7 | 0.3 | 0.2 | 0.7 | 0.4 | 0.7 | 1.0 | 0.6 |
| Tauro-conjugated | 0.5 | 0.2 | 1.3 | 0.1 | 0.1 | 0.8 | 0.1 | 0.5 | 0.2 | 0.3 |
| **Sulfated** | 0.2 | 0.6 | 0.4 | 0.6 | 0.1 | 1.0 | 1.2 | 0.2 | 0.2 | 0.3 |
| *UDCAs* | | | | | | | | | | |
| **UDCA** | 0.4 | 0.8 | 0.3 | 0.7 | 0.4 | 3.2 | 2.5 | 0.3 | 0.2 | 0.4 |
| GUDCA | 0.1 | 0.3 | 0 | 0 | 0.1 | 0.1 | 0 | 0.5 | 0 | 0 |
| TUDCA | 0 | 0 | 0 | 0 | 0 | 0.1 | 0 | 0.1 | 0 | 0 |
| UDCA-3S | 0 | 0.1 | 0.1 | 0 | 0 | 0.1 | 0 | 0 | 0 | 0.2 |
| GUDCA-3S | 0 | 0 | 0 | 0 | 0 | 0 | 0 | 0 | 0 | 0 |
| TUDCA-3S | 0 | 0 | 0 | 0 | 0 | 0 | 0 | 0 | 0 | 0 |
| **Total** | 0.5 | 1.2 | 0.3 | 0.7 | 0.5 | 3.4 | 2.5 | 1.0 | 0.2 | 0.6 |

| **Table S2b – Median of bile acid total concentration (nmol/g) and subtypes as percent of the total bile acid content (%) measured at baseline compared between the donors used in the RCT** (D1, D8-10) **versus all donors/healthy controls** (D1-D10) | | | |
| --- | --- | --- | --- |
|  | **Donors in RCT** | **All donors** | **p-value** |
| **Total concentration, nmol/g** | 2701 | 2729 | 0.95 |
| **Total primary BAs, %** | 11.25 | 12.90 | 0.84 |
| CA | 2.20 | 2.76 | 0.62 |
| CDCA | 1.95 | 2.25 | 0.37 |
| TCA | 1.50 | 1.18 | 0.94 |
| **Total secondary BAs, %** | 88.20 | 85.30 | 0.54 |
| DCA | 49.00 | 47.60 | 0.73 |
| LCA | 37.65 | 37.68 | 0.95 |
| **Total UCDAs, %** | 0.55 | 0.65 | 0.54 |

| **Table S3 - Median total concentration of bile acids before and after treatments, nmol/g** | | | | | | |
| --- | --- | --- | --- | --- | --- | --- |
|  | **rCDI (all)** | **FMT** | **RBT** | **Vancomycin** | **Donors** | **rCDI vs. donors, p** |
| **Baseline** | 11627 | 12713 | 10428 | 13503 | 2729 | 0.0001 |
| **Day 90** | 4486 | 4158 | 5820 | 3821 |  | 0.12 |
| **Day 180** | 5744 | 5763 | 6012 | 5476 |  | 0.06 |

| **Table S4 - Abundance of known bile acid transforming bacteria, data from 16S rDNA gene sequencing**  ******* *Sample excluded due an unreliable low number of reads* | | | | | | | | | | |
| --- | --- | --- | --- | --- | --- | --- | --- | --- | --- | --- |
|  | **Abundance, count**  i.e. number of reads of the species | | | | | **Relative abundance, percent (%)**  **i**.e. number of reads of the species out of the total number of reads in percent (%) | | | | |
| **Sample** | **C. hiranonis** | **C. hylemonae** | **C.**  **leptum** | **C. scindens** | **C.**  **sordellii** | **C. hiranonis** | **C. hylemonae** | **C.**  **leptum** | **C. scindens** | **C.**  **sordellii** |
| **A_baseline** | 0 | 0 | 0 | 0 | 0 | 0,000 | 0,000 | 0,000 | 0,000 | 0,000 |
| **A_day3** | 0 | 0 | 0 | 0 | 0 | 0,000 | 0,000 | 0,000 | 0,000 | 0,000 |
| **A_day7** | 0 | 0 | 0 | 0 | 0 | 0,000 | 0,000 | 0,000 | 0,000 | 0,000 |
| **A_day14** | 0 | 0 | 0 | 0 | 0 | 0,000 | 0,000 | 0,000 | 0,000 | 0,000 |
| **A_day30** | 0 | 0 | 0 | 0 | 0 | 0,000 | 0,000 | 0,000 | 0,000 | 0,000 |
| **A_Day90** | 0 | 0 | 0 | 0 | 0 | 0,000 | 0,000 | 0,000 | 0,000 | 0,000 |
| **A_Day180** | 0 | 0 | 0 | 0 | 0 | 0,000 | 0,000 | 0,000 | 0,000 | 0,000 |
| **B_baseline** | 0 | 0 | 0 | 0 | 0 | 0,000 | 0,000 | 0,000 | 0,000 | 0,000 |
| **B_day3** | 4 | 0 | 0 | 0 | 0 | 0,004 | 0,000 | 0,000 | 0,000 | 0,000 |
| **B_day7** | 0 | 0 | 0 | 0 | 0 | 0,000 | 0,000 | 0,000 | 0,000 | 0,000 |
| **B_day14** | 0 | 48 | 0 | 0 | 0 | 0,000 | 0,060 | 0,000 | 0,000 | 0,000 |
| **B_day30** | 0 | 86 | 0 | 0 | 0 | 0,000 | 0,105 | 0,000 | 0,000 | 0,000 |
| **B_day90** | 0 | 0 | 0 | 428 | 0 | 0,000 | 0,000 | 0,000 | 0,568 | 0,000 |
| **B_day180** | 0 | 0 | 0 | 37 | 0 | 0,000 | 0,000 | 0,000 | 0,045 | 0,000 |
| **C_baseline** | 0 | 0 | 0 | 0 | 0 | 0,000 | 0,000 | 0,000 | 0,000 | 0,000 |
| **C_day3** | 0 | 0 | 0 | 27 | 0 | 0,000 | 0,000 | 0,000 | 0,031 | 0,000 |
| **C_day7** | 0 | 27 | 0 | 54 | 0 | 0,000 | 0,025 | 0,000 | 0,050 | 0,000 |
| **C_day14** | 0 | 0 | 0 | 54 | 0 | 0,000 | 0,000 | 0,000 | 0,053 | 0,000 |
| **C_day30** | 0 | 0 | 0 | 356 | 0 | 0,000 | 0,000 | 0,000 | 0,364 | 0,000 |
| **C_day90** | 0 | 489 | 0 | 143 | 0 | 0,000 | 0,521 | 0,000 | 0,152 | 0,000 |
| **C_day180** | 0 | 786 | 0 | 87 | 0 | 0,000 | 0,846 | 0,000 | 0,094 | 0,000 |
| **D_baseline** | 0 | 0 | 0 | 0 | 0 | 0,000 | 0,000 | 0,000 | 0,000 | 0,000 |
| **D_day3** | 0 | 0 | 0 | 0 | 0 | 0,000 | 0,000 | 0,000 | 0,000 | 0,000 |
| **D_day7** | 0 | 0 | 0 | 0 | 0 | 0,000 | 0,000 | 0,000 | 0,000 | 0,000 |
| **D_day14** | 0 | 0 | 0 | 0 | 0 | NA | NA | NA | NA | NA |
| **D_day30** | 0 | 0 | 0 | 0 | 0 | 0,000 | 0,000 | 0,000 | 0,000 | 0,000 |
| **D_day90** | 0 | 0 | 0 | 0 | 0 | 0,000 | 0,000 | 0,000 | 0,000 | 0,000 |
| **D_day180** | 0 | 0 | 0 | 0 | 0 | 0,000 | 0,000 | 0,000 | 0,000 | 0,000 |
| **E_baseline** | 0 | 0 | 0 | 0 | 0 | 0,000 | 0,000 | 0,000 | 0,000 | 0,000 |
| **E_day3** | 0 | 0 | 0 | 0 | 0 | 0,000 | 0,000 | 0,000 | 0,000 | 0,000 |
| **E_day7** | 0 | 0 | 0 | 0 | 0 | 0,000 | 0,000 | 0,000 | 0,000 | 0,000 |
| **E_day14** | 0 | 0 | 0 | 234 | 0 | 0,000 | 0,000 | 0,000 | 0,269 | 0,000 |
| **E_day30** | 0 | 0 | 0 | 0 | 0 | 0,000 | 0,000 | 0,000 | 0,000 | 0,000 |
| **E_day90** | 0 | 0 | 0 | 23 | 0 | 0,000 | 0,000 | 0,000 | 0,037 | 0,000 |
| **E_day180** | 0 | 0 | 0 | 0 | 0 | 0,000 | 0,000 | 0,000 | 0,000 | 0,000 |
|  | **Abundance, count**  i.e. number of reads of the species | | | | | **Relative abundance, percent (%)**  **i**.e. number of reads of the species out of the total number of reads in percent (%) | | | | |
| **Sample** | **C. hiranonis** | **C. hylemonae** | **C.**  **leptum** | **C. scindens** | **C.**  **sordellii** | **C. hiranonis** | **C. hylemonae** | **C.**  **leptum** | **C. scindens** | **C.**  **sordellii** |
| **F_baseline** | 0 | 0 | 0 | 0 | 0 | 0,000 | 0,000 | 0,000 | 0,000 | 0,000 |
| **F_day3** | 0 | 0 | 0 | 0 | 0 | 0,000 | 0,000 | 0,000 | 0,000 | 0,000 |
| **F_day7** | 0 | 0 | 0 | 0 | 0 | 0,000 | 0,000 | 0,000 | 0,000 | 0,000 |
| **F_day14** | 0 | 0 | 0 | 0 | 0 | 0,000 | 0,000 | 0,000 | 0,000 | 0,000 |
| **F_day30** | 0 | 0 | 0 | 0 | 0 | 0,000 | 0,000 | 0,000 | 0,000 | 0,000 |
| **F_day90*** | 0 | 0 | 0 | 0 | 0 | 0,000 | 0,000 | 0,000 | 0,000 | 0,000 |
| **F_day180** | 0 | 0 | 0 | 0 | 0 | 0,000 | 0,000 | 0,000 | 0,000 | 0,000 |
| **G_baseline** | 0 | 0 | 0 | 0 | 0 | 0,000 | 0,000 | 0,000 | 0,000 | 0,000 |
| **G_day3** | 0 | 0 | 0 | 0 | 0 | 0,000 | 0,000 | 0,000 | 0,000 | 0,000 |
| **G_day7** | 0 | 0 | 0 | 0 | 0 | 0,000 | 0,000 | 0,000 | 0,000 | 0,000 |
| **G_day14** | 0 | 20 | 0 | 0 | 0 | 0,000 | 0,025 | 0,000 | 0,000 | 0,000 |
| **G_day30** | 0 | 0 | 2053 | 0 | 0 | 0,000 | 0,000 | 2,229 | 0,000 | 0,000 |
| **G_day90** | 0 | 0 | 0 | 14 | 0 | 0,000 | 0,000 | 0,000 | 0,024 | 0,000 |
| **G_day180** | 0 | 0 | 0 | 0 | 0 | 0,000 | 0,000 | 0,000 | 0,000 | 0,000 |
| **H_baseline** | 0 | 0 | 0 | 0 | 0 | 0,000 | 0,000 | 0,000 | 0,000 | 0,000 |
| **H_day3** | 0 | 74 | 0 | 0 | 0 | 0,000 | 0,079 | 0,000 | 0,000 | 0,000 |
| **I_baseline** | 0 | 0 | 0 | 0 | 0 | 0,000 | 0,000 | 0,000 | 0,000 | 0,000 |
| **I_day3** | 0 | 0 | 0 | 0 | 0 | 0,000 | 0,000 | 0,000 | 0,000 | 0,000 |
| **I_day7** | 0 | 0 | 0 | 0 | 0 | 0,000 | 0,000 | 0,000 | 0,000 | 0,000 |
| **I_day14** | 0 | 0 | 0 | 11 | 0 | 0,000 | 0,000 | 0,000 | 0,017 | 0,000 |
| **I_day30** | 0 | 0 | 0 | 28 | 0 | 0,000 | 0,000 | 0,000 | 0,036 | 0,000 |
| **I_day90** | 0 | 0 | 0 | 12 | 0 | 0,000 | 0,000 | 0,000 | 0,019 | 0,000 |
| **I_day180** | 0 | 0 | 0 | 0 | 0 | 0,000 | 0,000 | 0,000 | 0,000 | 0,000 |
| **J_baseline** | 0 | 0 | 0 | 0 | 0 | 0,000 | 0,000 | 0,000 | 0,000 | 0,000 |
| **J_day3** | 0 | 0 | 0 | 0 | 0 | 0,000 | 0,000 | 0,000 | 0,000 | 0,000 |
| **J_day7** | 0 | 4315 | 0 | 0 | 0 | 0,000 | 4,867 | 0,000 | 0,000 | 0,000 |
| **J_day14** | 0 | 0 | 0 | 0 | 0 | 0,000 | 0,000 | 0,000 | 0,000 | 0,000 |
| **J_day30** | 0 | 0 | 0 | 0 | 0 | 0,000 | 0,000 | 0,000 | 0,000 | 0,000 |
| **J_day90** | 0 | 0 | 0 | 16 | 0 | 0,000 | 0,000 | 0,000 | 0,022 | 0,000 |
| **J_day180** | 0 | 0 | 0 | 0 | 0 | 0,000 | 0,000 | 0,000 | 0,000 | 0,000 |
| **K_baseline** | 0 | 0 | 0 | 0 | 0 | 0,000 | 0,000 | 0,000 | 0,000 | 0,000 |
| **K_day3** | 0 | 156 | 0 | 0 | 0 | 0,000 | 0,222 | 0,000 | 0,000 | 0,000 |
| **K_day7** | 0 | 0 | 0 | 247 | 0 | 0,000 | 0,000 | 0,000 | 0,273 | 0,000 |
| **K_day14** | 0 | 0 | 0 | 0 | 0 | NA | NA | NA | NA | NA |
| **K_day30** | 0 | 0 | 0 | 49 | 0 | 0,000 | 0,000 | 0,000 | 0,044 | 0,000 |
| **K_day90** | 0 | 357 | 0 | 40 | 0 | 0,000 | 0,429 | 0,000 | 0,048 | 0,000 |
| **K_day180** | 0 | 2395 | 0 | 233 | 0 | 0,000 | 2,462 | 0,000 | 0,240 | 0,000 |
|  | **Abundance, count**  i.e. number of reads of the species | | | | | **Relative abundance, percent (%)**  **i**.e. number of reads of the species out of the total number of reads in percent (%) | | | | |
| **Sample** | **C. hiranonis** | **C. hylemonae** | **C.**  **leptum** | **C. scindens** | **C.**  **sordellii** | **C. hiranonis** | **C. hylemonae** | **C.**  **leptum** | **C. scindens** | **C.**  **sordellii** |
| **L_baseline*** | 0 | 0 | 0 | 0 | 0 | 0,000 | 0,000 | 0,000 | 0,000 | 0,000 |
| **L_day3** | 0 | 0 | 0 | 0 | 0 | 0,000 | 0,000 | 0,000 | 0,000 | 0,000 |
| **L_day7*** | 0 | 0 | 0 | 0 | 0 | 0,000 | 0,000 | 0,000 | 0,000 | 0,000 |
| **L_day14** | 0 | 0 | 0 | 0 | 0 | 0,000 | 0,000 | 0,000 | 0,000 | 0,000 |
| **L_day30*** | 0 | 0 | 0 | 0 | 0 | 0,000 | 0,000 | 0,000 | 0,000 | 0,000 |
| **L_day90** | 0 | 300 | 0 | 280 | 0 | 0,000 | 0,439 | 0,000 | 0,410 | 0,000 |
| **L_day180** | 0 | 758 | 0 | 128 | 0 | 0,000 | 1,259 | 0,000 | 0,213 | 0,000 |
| **M_baseline** | 0 | 0 | 0 | 0 | 0 | 0,000 | 0,000 | 0,000 | 0,000 | 0,000 |
| **M_day3*** | 0 | 0 | 0 | 0 | 0 | 0,000 | 0,000 | 0,000 | 0,000 | 0,000 |
| **M_day7** | 0 | 0 | 0 | 0 | 0 | 0,000 | 0,000 | 0,000 | 0,000 | 0,000 |
| **M_day14** | 0 | 0 | 0 | 270 | 0 | 0,000 | 0,000 | 0,000 | 0,457 | 0,000 |
| **M_day30** | 0 | 0 | 0 | 53 | 0 | 0,000 | 0,000 | 0,000 | 0,123 | 0,000 |
| **M_day90** | 0 | 0 | 0 | 41 | 0 | 0,000 | 0,000 | 0,000 | 0,088 | 0,000 |
| **M_day180** | 0 | 0 | 0 | 11 | 0 | 0,000 | 0,000 | 0,000 | 0,022 | 0,000 |
| **N_baseline** | 0 | 0 | 0 | 0 | 0 | 0,000 | 0,000 | 0,000 | 0,000 | 0,000 |
| **N_day3** | 0 | 0 | 0 | 0 | 0 | 0,000 | 0,000 | 0,000 | 0,000 | 0,000 |
| **N_day7** | 0 | 0 | 0 | 0 | 0 | 0,000 | 0,000 | 0,000 | 0,000 | 0,000 |
| **N_day14** | 0 | 0 | 0 | 0 | 0 | 0,000 | 0,000 | 0,000 | 0,000 | 0,000 |
| **O_baseline** | 0 | 0 | 0 | 0 | 0 | 0,000 | 0,000 | 0,000 | 0,000 | 0,000 |
| **O_day3*** | 0 | 0 | 0 | 0 | 0 | 0,000 | 0,000 | 0,000 | 0,000 | 0,000 |
| **O_day7*** | 0 | 0 | 0 | 0 | 0 | 0,000 | 0,000 | 0,000 | 0,000 | 0,000 |
| **O_day14*** | 0 | 0 | 0 | 0 | 0 | 0,000 | 0,000 | 0,000 | 0,000 | 0,000 |
| **O_day30*** | 0 | 0 | 0 | 0 | 0 | 0,000 | 0,000 | 0,000 | 0,000 | 0,000 |
| **O_day90*** | 0 | 0 | 0 | 0 | 0 | 0,000 | 0,000 | 0,000 | 0,000 | 0,000 |
| **O_day180*** | 0 | 0 | 0 | 0 | 0 | 0,000 | 0,000 | 0,000 | 0,000 | 0,000 |
| **P_baseline** | 1 | 0 | 0 | 0 | 0 | 0,003 | 0,000 | 0,000 | 0,000 | 0,000 |
| **P_day3** | 0 | 374 | 0 | 0 | 0 | 0,000 | 0,539 | 0,000 | 0,000 | 0,000 |
| **P_day7** | 0 | 34 | 0 | 0 | 0 | 0,000 | 0,036 | 0,000 | 0,000 | 0,000 |
| **P_day14** | 1 | 1601 | 0 | 222 | 0 | 0,002 | 2,594 | 0,000 | 0,360 | 0,000 |
| **P_day30** | 0 | 0 | 0 | 810 | 0 | 0,000 | 0,000 | 0,000 | 1,018 | 0,000 |
| **P_day90** | 0 | 0 | 0 | 1565 | 0 | 0,000 | 0,000 | 0,000 | 2,016 | 0,000 |
| **P_day180** | 0 | 116 | 0 | 1774 | 0 | 0,000 | 0,147 | 0,000 | 2,250 | 0,000 |
| **Q_baseline*** | 0 | 0 | 0 | 0 | 0 | 0,000 | 0,000 | 0,000 | 0,000 | 0,000 |
| **Q_day3** | 0 | 0 | 0 | 0 | 0 | 0,000 | 0,000 | 0,000 | 0,000 | 0,000 |
| **Q_day7** | 0 | 0 | 0 | 0 | 0 | 0,000 | 0,000 | 0,000 | 0,000 | 0,000 |
| **Q_day14** | 0 | 0 | 0 | 1172 | 0 | 0,000 | 0,000 | 0,000 | 0,997 | 0,000 |
| **Q_day30** | 0 | 0 | 0 | 212 | 0 | 0,000 | 0,000 | 0,000 | 0,145 | 0,000 |
| **Q_day90** | 0 | 0 | 0 | 0 | 0 | 0,000 | 0,000 | 0,000 | 0,000 | 0,000 |
| **Q_day180** | 0 | 0 | 0 | 0 | 0 | 0,000 | 0,000 | 0,000 | 0,000 | 0,000 |
|  | **Abundance, count**  i.e. number of reads of the species | | | | | **Relative abundance, percent (%)**  **i**.e. number of reads of the species out of the total number of reads in percent (%) | | | | |
| **Sample** | **C. hiranonis** | **C. hylemonae** | **C.**  **leptum** | **C. scindens** | **C.**  **sordellii** | **C. hiranonis** | **C. hylemonae** | **C.**  **leptum** | **C. scindens** | **C.**  **sordellii** |
| **R_baseline** | 0 | 0 | 0 | 0 | 0 | 0,000 | 0,000 | 0,000 | 0,000 | 0,000 |
| **R_day3** | 0 | 0 | 0 | 0 | 0 | 0,000 | 0,000 | 0,000 | 0,000 | 0,000 |
| **R_day7*** | 0 | 0 | 0 | 0 | 0 | 0,000 | 0,000 | 0,000 | 0,000 | 0,000 |
| **R_day14*** | 0 | 0 | 0 | 0 | 0 | 0,000 | 0,000 | 0,000 | 0,000 | 0,000 |
| **R_day30** | 0 | 0 | 0 | 0 | 0 | 0,000 | 0,000 | 0,000 | 0,000 | 0,000 |
| **R_day90** | 0 | 0 | 0 | 0 | 0 | 0,000 | 0,000 | 0,000 | 0,000 | 0,000 |
| **R_day180*** | 0 | 0 | 0 | 0 | 0 | 0,000 | 0,000 | 0,000 | 0,000 | 0,000 |
| **S_baseline** | 0 | 0 | 0 | 0 | 0 | 0,000 | 0,000 | 0,000 | 0,000 | 0,000 |
| **S_day3** | 0 | 0 | 0 | 0 | 5 | 0,000 | 0,000 | 0,000 | 0,000 | 0,008 |
| **S_day7** | 0 | 0 | 0 | 0 | 0 | 0,000 | 0,000 | 0,000 | 0,000 | 0,000 |
| **S_day14** | 0 | 0 | 0 | 0 | 0 | 0,000 | 0,000 | 0,000 | 0,000 | 0,000 |
| **S_day30** | 0 | 0 | 0 | 0 | 0 | 0,000 | 0,000 | 0,000 | 0,000 | 0,000 |
| **S_day90** | 0 | 0 | 0 | 0 | 0 | 0,000 | 0,000 | 0,000 | 0,000 | 0,000 |
| **S_day180** | 0 | 133 | 0 | 0 | 0 | 0,000 | 0,262 | 0,000 | 0,000 | 0,000 |
| **T_baseline*** | 0 | 0 | 0 | 0 | 0 | 0,000 | 0,000 | 0,000 | 0,000 | 0,000 |
| **T_day3** | 0 | 0 | 0 | 0 | 0 | 0,000 | 0,000 | 0,000 | 0,000 | 0,000 |
| **T_day7** | 0 | 0 | 0 | 0 | 0 | 0,000 | 0,000 | 0,000 | 0,000 | 0,000 |
| **T_day14** | 0 | 0 | 0 | 0 | 0 | 0,000 | 0,000 | 0,000 | 0,000 | 0,000 |
| **T_day30** | 0 | 0 | 0 | 25 | 0 | 0,000 | 0,000 | 0,000 | 0,085 | 0,000 |
| **T_day90** | 0 | 0 | 0 | 0 | 0 | 0,000 | 0,000 | 0,000 | 0,000 | 0,000 |
| **T_day180** | 0 | 0 | 0 | 35 | 0 | 0,000 | 0,000 | 0,000 | 0,049 | 0,000 |
| **U_baseline** | 0 | 168 | 0 | 0 | 0 | 0,000 | 0,220 | 0,000 | 0,000 | 0,000 |
| **U_day3** | 0 | 0 | 0 | 0 | 0 | 0,000 | 0,000 | 0,000 | 0,000 | 0,000 |
| **U_day14** | 0 | 0 | 0 | 0 | 0 | 0,000 | 0,000 | 0,000 | 0,000 | 0,000 |
| **D1** | 0 | 0 | 0 | 0 | 0 | 0,000 | 0,000 | 0,000 | 0,000 | 0,000 |
| **D2** | 0 | 0 | 0 | 0 | 0 | 0,000 | 0,000 | 0,000 | 0,000 | 0,000 |
| **D3** | 0 | 0 | 0 | 0 | 0 | 0,000 | 0,000 | 0,000 | 0,000 | 0,000 |
| **D4** | 0 | 0 | 0 | 0 | 0 | 0,000 | 0,000 | 0,000 | 0,000 | 0,000 |
| **D5** | 0 | 0 | 0 | 0 | 0 | 0,000 | 0,000 | 0,000 | 0,000 | 0,000 |
| **D6** | 0 | 0 | 0 | 0 | 0 | 0,000 | 0,000 | 0,000 | 0,000 | 0,000 |
| **D7** | 0 | 0 | 0 | 0 | 0 | 0,000 | 0,000 | 0,000 | 0,000 | 0,000 |
| **D8** | 0 | 0 | 0 | 0 | 0 | 0,000 | 0,000 | 0,000 | 0,000 | 0,000 |
| **D9*** | 0 | 0 | 0 | 0 | 0 | 0,000 | 0,000 | 0,000 | 0,000 | 0,000 |
| **D10** | 0 | 0 | 0 | 0 | 0 | 0,000 | 0,000 | 0,000 | 0,000 | 0,000 |

**
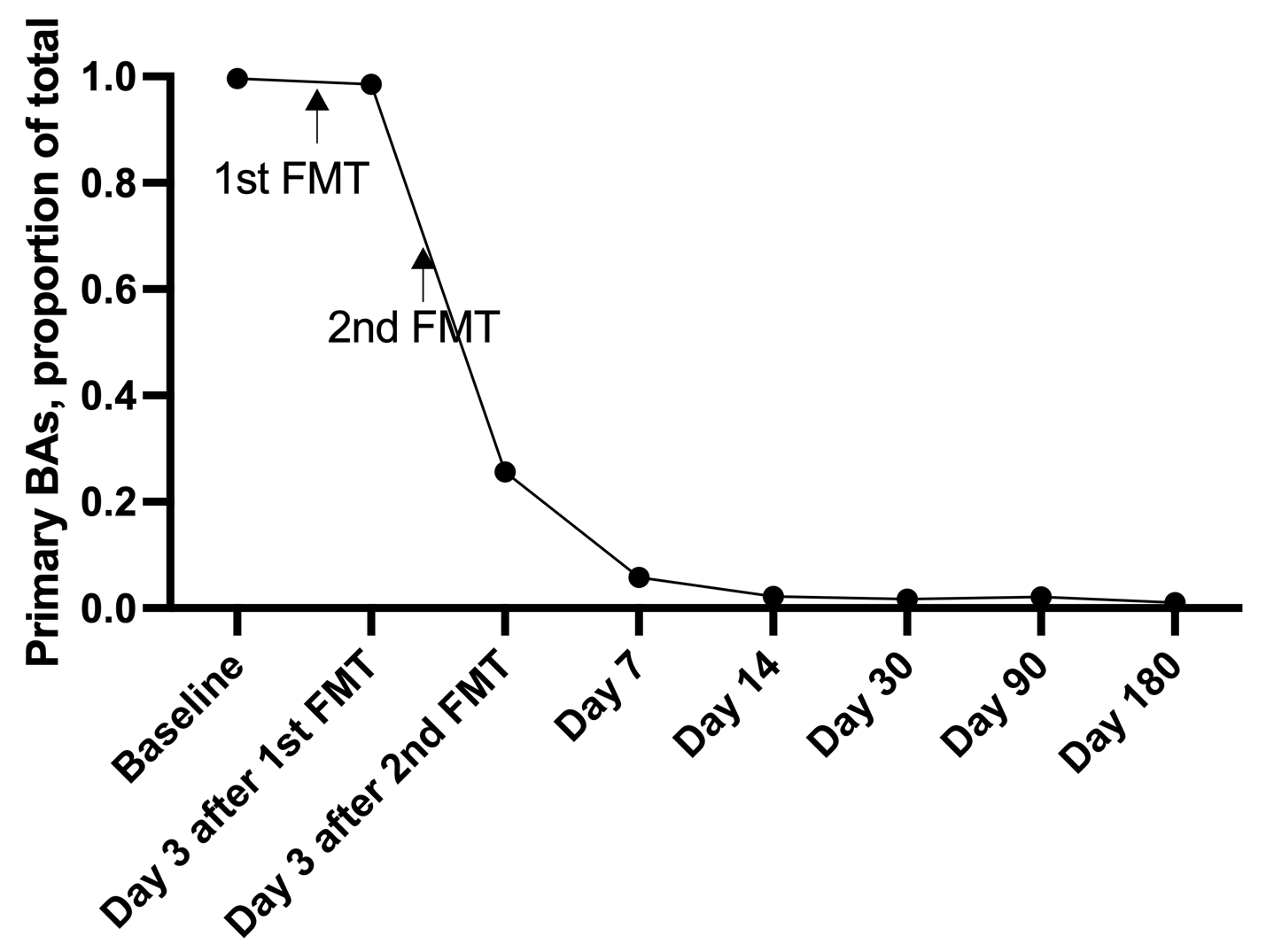
**

**Figure S1.** The proportion of primary bile acids of the total content of bile acids in samples from participant K after treatment with respectively the first unsuccessful FMT and the second successful FMT with a different donor.

**Figure S2**. The proportion of conjugated (left side) and sulphated (right side) bile acids of the total content of bile acids over time after treatment in individuals after RBT (top), FMT (middle) and vancomycin monotherapy (bottom).

Note that the sample taken just prior to RBT/FMT (day 0) and the sample taken the day after the last vancomycin-intake (day 1) is included. Dashed lines: Participants experiencing failure of treatment; Solid lines: Participants with clinical cure at 90-days follow-up. The median level for healthy donors is shown in each plot for comparison.

BAs: Bile acids.

**Figure S3**. The proportions of the main subtypes of primary bile acids (top) and secondary bile acids (bottom) of the total content of bile acids over time after treatment with FMT. Note that the sample taken just prior to FMT is included in this graph (day 0). The median level for healthy donors is shown in each plot for comparison.

CA: Cholic acid; CDCA: Chenodeoxycholic acid; GCA: Glyco-conjugated cholic acid; GCDCA: Glyco-conjugated chenodeoxycholic acid; LCA: Lithocholic acid; DCA: Deoxycholic acid.

**Figure S4**. The proportions of the main subtypes of primary bile acids (top) and secondary bile acids (bottom) of the total content of bile acids over time after treatment with RBT. Note that the sample taken just prior to RBT is included in this graph (day 0). Dashed lines: Participants experiencing failure of treatment; Solid lines: Participants with clinical cure at 90-days follow-up. The median level for healthy donors is shown in each plot for comparison.

CA: Cholic acid; CDCA: Chenodeoxycholic acid; GCA: Glyco-conjugated cholic acid; GCDCA: Glyco-conjugated chenodeoxycholic acid; LCA: Lithocholic acid; DCA: Deoxycholic acid.

**Figure S5**. The proportions of the main subtypes of primary bile acids (top) and secondary bile acids (bottom) of the total content of bile acids over time after treatment with vancomycin monotherapy. Note that the sample taken on the day after last vancomycin-intake (day 1). Dashed lines: Participants experiencing failure of treatment; Solid lines: Participants with clinical cure at 90-days follow-up The median level for healthy donors is shown in each plot for comparison.

CA: Cholic acid; CDCA: Chenodeoxycholic acid; GCA: Glyco-conjugated cholic acid; GCDCA: Glyco-conjugated chenodeoxycholic acid; LCA: Lithocholic acid; DCA: Deoxycholic acid.
